# Integrating the Principles of Evidence Based Medicine and Evidence Based Public Health: Impact on the Quality of Patient Care and Hospital Readmission Rates in Jordan

**DOI:** 10.5334/ijic.2436

**Published:** 2016-08-31

**Authors:** Mohammad S. Alyahya, Heba H. Hijazi, Hussam A. Alshraideh, Mohammad Aser Alsharman, Rabah Al Abdi, Heather Lea Harvey

**Affiliations:** 1 Department of Health Management and Policy, Faculty of Medicine Jordan University of Science and Technology, P.O. Box: 3030, Irbid 22110, Jordan; 2 Chairman of the Department of Health Management and Policy, Faculty of Medicine Jordan University of Science and Technology, P.O. Box 3030, Irbid 22110, Jordan; 3 Industrial Engineering Jordan University of Science and Technology, P.O. Box: 3030, Irbid 22110, Jordan; 4 King Abdullah University Hospital/Irbid/Jordan; 5 Biomedical Engineering Jordan University of Science and Technology, P.O. Box: 3030, Irbid 22110, Jordan

**Keywords:** Hospital readmissions, avoidable readmissions, evidence based medicine, evidence based public health, behavioural risk factors

## Abstract

**Introduction:**

Hospital readmissions impose not only an extra burden on health care systems but impact patient health outcomes. Identifying modifiable behavioural risk factors that are possible causes of potentially avoidable readmissions can lower readmission rates and healthcare costs.

**Methods:**

Using the core principles of evidence based medicine and public health, the purpose of this study was to develop a heuristic guide that could identify what behavioural risk factors influence hospital readmissions through adopting various methods of analysis including regression models, t-tests, data mining, and logistic regression. This study was a retrospective cohort review of internal medicine patients admitted between December 1, 2012 and December 31, 2013 at King Abdullah University Hospital, in Jordan.

**Results:**

29% of all hospitalized patients were readmitted during the study period. Among all readmissions, 44% were identified as potentially avoidable. Behavioural factors including smoking, unclear follow-up and discharge planning, and being non-compliant with treatment regimen as well as discharge against medical advice were all associated with increased risk of avoidable readmissions.

**Conclusion:**

Implementing evidence based health programs that focus on modifiable behavioural risk factors for both patients and clinicians would yield a higher response in terms of reducing potentially avoidable readmissions, and could reduce direct medical costs.

## Introduction

Hospital readmission is a global health-care problem that poses a financial burden to healthcare systems [1,2]. In Jordan, the average cost per patient for an internal medicine readmission is approximately $2,000. For decades, readmissions have been considered a persistent endemic to healthcare systems worldwide, while avoidable ones are becoming a growing pandemic [3,4,5]. From a public health perspective, how to prevent or reduce avoidable hospital readmissions should be seen as a primary health issue that needs to be better understood, so that effective methods of prevention can be adopted to reduce the burden on the healthcare system as well as improve patient safety and clinical outcomes.

Substantial efforts have been devoted to determining what factors may be associated with early rehospitalisation. Patient characteristics such as age, main diagnosis, co-morbidities, and race have been studied and shown to be well-known risk factors [6,7,8,9]. Patients who receive inappropriate or inadequate medical assessment and treatment are more likely to be readmitted, indicating not only are patient characteristics but quality of care is associated with readmission [10]. But, how other clinical factors impact readmission has been elusive and the results vary based on specific medical indicators and clinical conditions [11,12]. While some criteria for readmissions are moderately predictable, and several prediction models have shown promising initiatives in forecasting the rates of patients at the greatest risk of readmission, their overall performance has been limited [13,14,15,16,17,18]. A systematic review conducted by Kansagara et al. [16] of different 26 predictive models argued that most of the available models that were developed for either comparative between hospitals or clinical purposes have limited ability to discriminate between readmissions and non-readmissions. Furthermore, only one validated prediction model was found that explicitly defined and determined avoidable readmissions. The authors added that hospital and health system-level factors such as post-discharge follow-up and coordination of care, which are not included in the existing models, could be associated with the risk of readmission.

Therefore, readmission is a multifaceted issue that still needs more in depth analysis to be better addressed. Using the core principles of evidence based medicine (EBM) and public health, the purpose of this study was to develop a heuristic guide that could identify what risk factors, and more specifically what behaviours influence early hospital readmissions using various methods of analysis, resulting in a model that would have greater “translation-transferability”, to reduce patient readmissions and healthcare costs, not only in Jordan but in other developing countries.

### Conceptual framework for hospital readmission

Patient readmission risk factors are typically categorised in two groups, patient-related and healthcare provider-related [19,20,21]. Patient factors include socio-demographics, health status and care, and patient behaviours [20,22,23]. Among the socio-demographic characteristics shown to impact readmission, patient age appears to have the most consistent relationship with risk of readmission [20,24,25,26]. Other demographic variables—such as gender, residential address, and marital status—are also potential predictors of readmission, however these are not avoidable or modifiable [8,17,27,28,29,30,31,32], as does the patient’s health insurance status [17,29,30]. Coinsurance is a predetermined specific percentage of money that patient pays for healthcare services, typically is due at the time of service, and then the insurance agency pays the rest [33]. Insurance status impacts health services utilization and health outcomes [34,35]. Several readmission risk models have shown that insurance status is as significant predictors of 30-day readmission [15,36,37,38]. The patient’s primary diagnosis, number of co-morbidities, number of prior hospitalisations during the last 12 months, and length of stay also influence readmission [9,17,20,39,40]. More critical though is that certain patient behaviours have been associated with the likelihood of readmission, but are avoidable. These include: discharge against medical advice, non-compliance with treatment, and smoking [16,41,42,43]. While the impact of smoking as a predictor of early readmission for chronic obstructive pulmonary disease (COPD) patients is known [44,45,46], it is association with readmission for other health conditions is not well studied.

**Figure 1:**
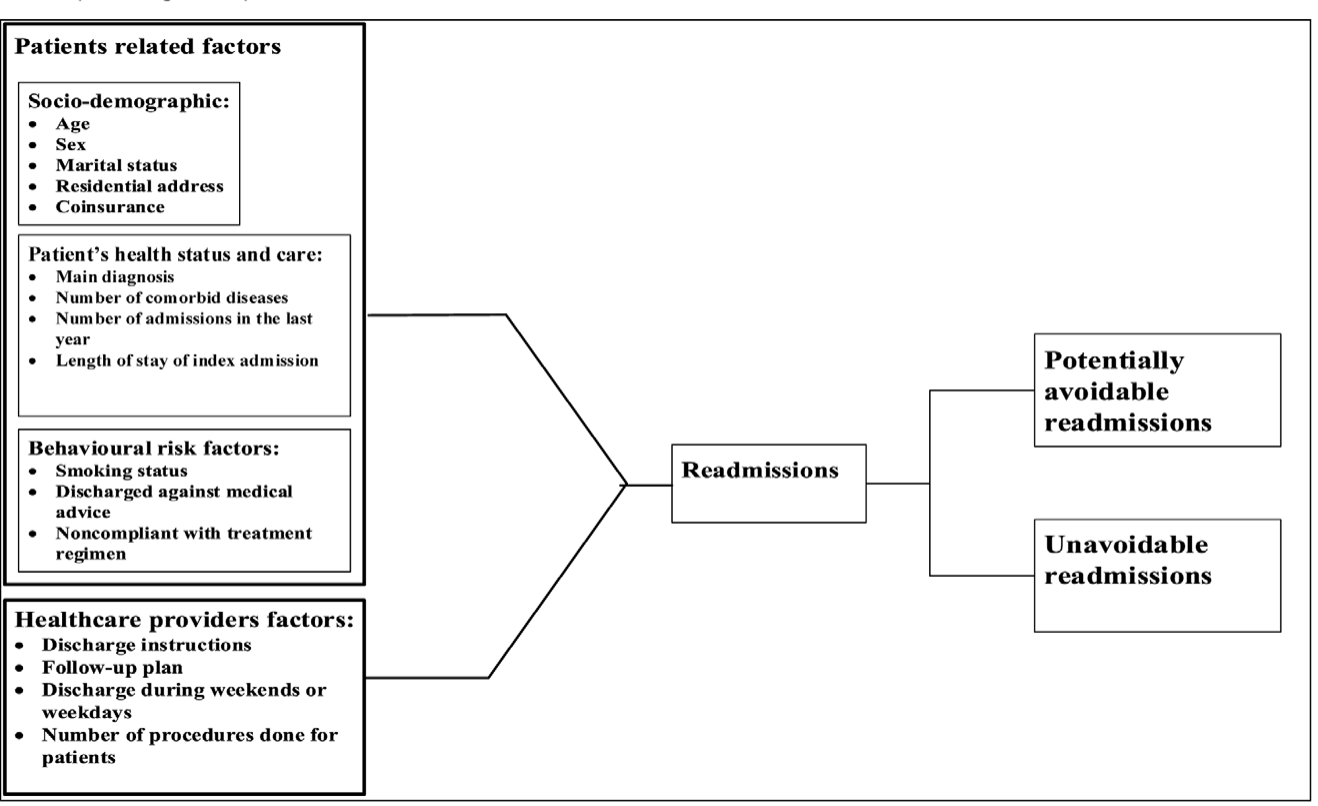
Conceptual Framework.

In addition to identifying patient variables, determining healthcare provider-related factors may guide quality improvement efforts, even when all the predictor factors are not well known [47,48,49]. From this perspective, healthcare providers need to understand that providing clear and complete discharge instructions and follow-up plans play a significant role in reducing the risk of patient readmission [21,50,51,52]. Although the number of procedures performed for the patient in the index admission might be an important predicting variable of readmission, yet the role of this factor is still not clear enough [39]. Moreover, it has also been argued that patients discharged during weekends are at greater risk of readmission. The potential reason behind this is that limited services usually provided and less senior staff, especially clinicians are available over weekends [6,22,53]. The time immediately after discharge is also very critical, and patients are very vulnerable [50,54]. All of these factors are intertwined and each plays a potential role in readmissions (See Figure 1).

### Evidence Based Medicine & Evidence Based Public Health

Applying EBM has become the gold standard in healthcare facilities around the world [55]. Since being introduced in 1992, the goal has been to “improve the quality of decision-making and healthcare delivery across the world” [56, p.209], while integrating not only clinical knowledge and skills but ideally the patient’s values [57,58]. Using evidence based programmes can improve practice outcomes while reducing dysfunctional outcomes [59]. Existing evidence indicates that EBM could reduce hospital readmissions, but there is no conclusive evidence on the effectiveness of such programmes and the results varied across healthcare systems [60,61,62]. However, EBM’s focus on treatment protocols has not always been as good at facilitating support for change in behavioural practice [55,63].

Implementing evidence based programmes could reduce negative perceptions about health programmes and policies while increasing adoption and compliance [64]. While, evidence based public health is more nuanced and in the past decade has tried to develop and evaluate standards based on research of preventative health programmes and services, to determine which types of prevention programmes are more successful and the translation of such programmes across populations [65]. Therefore, combining these two innovative approaches could serve as a valuable means of reducing hospital readmissions for behavioural risk factors.

Over the past 20 years numerous models and theories including: Stages of Change by Procaskha [66], Bandura’s Social Cognitive Model [67], to Green’s Precede/Proceed Model [68] have been proposed to try and explain health behaviours. For years the model used to develop behavioural modification programmes was often left to the programme developers’ choice and preference, if any theory was applied at all. But more recently as evidence based public health as matured, Bully et al. [69] showed that different theoretical models have better success dependent on the health behaviour (i.e. diet, smoking, and physical activity). Furthermore, behavioural change programmes including those related to diet, smoking cessation and physical activity have been shown to benefit patients after being hospitalised [70].

Unfortunately, hospitals often pay the least attention to lifestyle factors and those behaviours that are modifiable, instead focusing on costs of diagnosis and treatment, and in the process do not give credence to preventable or avoidable factors [69]. While some hospital readmissions are planned and unavoidable, the scope of this study was to look at factors influencing avoidable readmissions to develop a guide for potential risk factors using clinical and non-clinical variables.

### Jordan

Jordan is a developing country in the upper-middle income range with an estimated population of 6.6 million, and with a health expenditure representing 7.2 % of its GDP-in 2013- [71,72]. This is high compared to other countries with similar means and stresses the economic system [73]. The health system in Jordan includes public, private, and international and non-governmental organisations, with 104 hospitals (totalling 18.9 hospital beds per 10,000) and almost half of the Jordanians rely on public health services [74]. Approximately 86% of Jordanians have some form of health insurance, while the government is striving towards a goal for universal coverage [75]. Chronic diseases are rising due to the adoption of western lifestyle behaviours including diet and exercise, and if not addressed hospital admissions and related readmissions will grow exponentially as the population ages.

## Methods

### Study design and setting

This study entailed a retrospective review of all internal medicine patients admitted between December 1, 2012 and December 31, 2013 at King Abdullah University Hospital (KAUH). KAUH is a multi-specialty hospital with 527 functional beds that could be expanded to 800 beds in an emergency. It is one of the leading teaching centres in Jordan that provides most major health specialties including surgery, medicine, mental health, oncology, maternity services, trauma services and more than 25 subspecialties. Although, the Ministry of Health regulates and supervises the sector as a whole, KAUH is financially and administratively independent. However, KAUH is a public hospital and its healthcare services are open to all Jordanians and non-Jordanians. It provides services to a population of more than one and half million people in four cities, reflects approximately one-quarter of the total population. The majority of individuals who seek treatment at KAUH are insured but the proportion of out-of-pocket costs through coinsurances varies widely. However, individuals who do not have health insurance or those who cannot afford their portion of the charges can obtain full health insurance coverage for free from the Royal Court, Prime Minister, or other public and private agencies. Thus, patients of KAUH represent a wide range of Jordanian patients.

All admissions were recorded from computerised medical records for each patient, and supplemented with paper-based medical records and telephone calls with patients or their families to obtain any missing data.

The study protocol was approved by the institutional review board at KAUH and the Deanship of Research at Jordan University of Science and Technology.

### Statistical design and data analysis

For all rehospitalised patients, early readmission was defined as a readmission within 30 days of discharge. Patient risk factors included; Socio-demographic (gender, marital status, coinsurance -being greater than or less than 20%-, distance between the hospital and patient’s home in Km –< 15, 15–29, 30–44, 45–59, > 60–’); patient’s health status and care (main diagnosis, number of comorbid diseases, number of admissions in the last year, and length of stay of index admission); and behavioural risk factors including smoking, discharge status (being with doctors’ orders or against medical advice (AMA)), and compliance with treatment plan. Hospital factors included: clear follow-up plans, discharge instructions, discharge during weekends or weekdays, and number of procedures done for patients during stay at the index admission. KAUH has been involved in the Joint Commission International (JCI) Programme since2007. The hospital was accredited for the first time by the JCI in 2009. JCI international standards for hospitals contain clear and specific measurable elements for follow-up plans and discharge instructions. Based on these measurable elements and standards, for each admission two of the authors reviewed the follow-up plans and discharge instructions which were obtained from the patient discharge summary and classified them as (complete and clear or incomplete and unclear) independently. Then research meetings were held regularly to verify accuracy, and any disagreement between the reviewers was resolved by discussion.

Various levels of statistical analysis were employed. At the first stage a specialised computerised algorithm, called Striving for Quality Level and Analysing of Patient Expenditures (SQLape) was used to determine readmissions that were avoidable. This algorithm took into account diagnostic and therapeutic processes of each index admission and readmission. For diagnostic codes, the International Classification of Diseases (ICD-10) was used, in conjunction with the Ninth Revision, Clinical Modification (ICD-9-CM) diagnosis and procedure codes. The SQLape classifies each readmission within 30 days of discharge as a potentially avoidable when it is linked to a previously established condition, or was unforeseen at the index admission. Compared with medical chart review, the true and false positive fractions for this algorithm were 96% and 4%. In details, readmissions were identified as avoidable if they were related to: 1) complications of treatment including surgical complications, healthcare complications, or obstetrical complications, 2) preventable conditions such as deep vein thrombosis, pulmonary embolism, and decubitus ulcer, 3) readmissions to preceding known conditions during the index admission, or 4) other healthcare complications such as dehydration, intrapartum haemorrhage, and endotoxic shock. In contrast, readmissions were identified unavoidable if they were: 1) planned readmissions such as readmission for elective surgery, labor and deliveries, chemotherapy or radiotherapy, leucopherese, bone marrow grafts, organ transplant and other special clinical procedures, 2) readmission for follow-up treatment and rehabilitation, 3) development of condition in a new body system unknown during the preceding hospital stay unless a known side effect of previously received treatment, or 4) readmissions because of trauma and diseases difficult to cure such as myelodysplastic syndrome, non-surgical intestinal adhesion, and acute bronchiolitis of nurseling [76,77,78].

Next, a multiple linear regression model was used to examine the relationships between hospital length of stay as dependent variable and all other factors including patients’ factors (socio-demographic, patients’ health status, and behavioural risk factors) as well as healthcare providers-related factors as independent variables. This was followed by t-tests for assessing the difference between the averages of the numerical factors with respect to gender, medical compliance and marital status, for both all readmissions and avoidable readmissions.

As an aim of this research study is to provide some kind of a Decision Support System (DSS) that can help healthcare providers in reducing avoidable readmissions, data mining techniques were employed. Data mining is a set of statistical and computer intelligence tools that are used to extract hidden information and build powerful prediction models for large datasets. Generally, data mining algorithms are used to solve classification and clustering problems [79]. Various classification algorithms are available, of which the most common are the C4.5 decision tree builder and JRip rule based classifier. Data mining provides the methodology and tools to transform large datasets into useful information for decision making. In healthcare, using data mining applications can greatly benefit all stakeholders. For instance, data mining can help health insurance companies to detect fraud and misuse, clinicians to identify effective treatments and best clinical practices, and patients to receive efficient care and avoid serious health problems [80].

Factors found to influence the type of readmission were fed into the C4.5 and JRip algorithms to build prediction models for mode of readmission as being avoidable or not. Model performance was assessed through the model prediction accuracy, which is how accurate the model was in predicting the mode of readmission. 10-fold cross validation was used to calculate the model accuracy. Both models provided similar prediction accuracy, with JRip model was preferred to the C4.5 as it provided fewer prediction rules for all cases considered in this study, and it demonstrated the best performance in terms of prediction accuracy, simplicity, and interpretability. WEKA software version 3.6 was used to build the prediction models.

The last stage involved a logistic regression model to determine if there was a risk between avoidable patient readmissions and the identified variables, and what specifically that risk measurement was. The association between both the continuous and binary independent variables with the main two outcomes was examined. These two outcomes were: 1) readmission status (readmitted/not readmitted), and 2) whether the readmission was avoidable or unavoidable. Binary independent variables included: gender, smoking, patient compliance, marital status, coinsurance (<20%/20+), discharge–decision (with medical advice/AMA), follow-up plan (clear/not clear), and discharge instruction (clear and documented/not), and the numeric values of: age, length of stay, readmission delay, previous admissions, number of procedures, and number of co-morbidities. All statistical analyses were performed using SPSS 17.0 (SPSS, Chicago, IL, USA).

## Results

Over the one year period, there were 5,273 consecutive internal medicine patient admissions. Excluded from the study were those patients who died (n = 155), stayed under 24 hours (n = 1,005), lived in another country (n = 98), transferred to another healthcare setting (n = 8), had a primary psychiatric or rehabilitation diagnosis (n = 6), or were discharged within the last 30 days of the study period.

Among the 3,962 eligible admissions, there were 1,157 (29%) early readmissions during the follow-up period. Using the SQLape algorithm, 506 (44%) of those readmissions were identified as potentially avoidable, representing 12.8% of all admissions. This rate may be considered high, but is comparable with international figures. In their systematic review, Walraven et al have found that the average proportion of readmissions deemed avoidable was 27.1% but varied significantly from 5% to 79% [43]. This wide variation could be due to patient selection, study setting and methodology, the variation in clinicians practice style, healthcare delivery systems, or the lack of consistency in the codes used to define different health conditions [27]. Thus, there is a need for developing consistent measures to identify potentially avoidable readmission [23]. However, in comparison with previous studies that examined the proportion of potentially avoidable readmissions occurred within 30 days of discharge from General Internal Medicine services these results are similar to previously reported rates of 39%, 40.5% and 53%, respectively, by Walsh et al. [81], Yam et al. [23], and Nahab et al. [82].

### Socio-demographic results

Much of the demographic data shows minimal difference between readmissions (n=1157) and avoidable readmissions (n=506). For all readmissions the mean age was 53.2, 46.9% were female, and 80.9% were married, compared to a mean age of 56.4, 44.7% female and 82.6% married for avoidable readmissions. Also, 86.1% (n=996) of all readmissions had less than 20% coinsurance, and on avoidable readmissions were 83.8% (n=424), while those with a coinsurance of 20% or greater only accounted for 13.9% (n=161) and in avoidable readmissions were 16.2% (n=82). However, the most common reason for readmission was due to malignant neoplasms 37% (n=428), but only 19% (n=96) of avoidable readmissions were malignant neoplasms related. However, the second largest group readmitted were those with circulatory diseases. They accounted for 13.8% of all readmissions (n= 160) and 19.8% of all avoidable readmissions (n= 100) making up the largest group of avoidable readmissions.

### Factors impacting readmissions

Similar results were found for both readmissions and avoidable readmissions. The multiple regression results showed no relation between the delay time of readmission as the dependent variable and any of the independent variables (number of co-morbidities, age, previous admissions, or number of procedures while hospitalised) with R^2^ between 0.002 and 0.004. However, length of stay in relation to age, previous admissions, and the number of procedures were all statistically significant (See Table 1). While age was inversely related to avoidable readmission, previous admissions and the number of procedures increased length of stay, meaning that a younger person, with more previous admissions and medical procedures would have longer hospital stay, explaining 44.9% and 41.3% of the variation in readmissions and avoidable readmissions, respectively.

**Table 1:** Multiple linear regression results for all readmissions and avoidable readmissions.

	All Readmissions		Avoidable Readmissions	

	Estimate	Significance	Estimate	Significance

(Intercept)	2.805	0.029	2.443	0.277
Age	–0.045	**0.027**	–0.045	**0.016**
Gender=Male	–0.212	0.516	–1.444	0.104
Marital Status=Single	0.859	0.173	2.494	0.117
Insurance Status= ≥20%	–0.067	0.154	0.853	0.181
Distance in Km=’> 60’	5.630	0.202	6.314	0.151
Distance in Km=’15–29’	–0.726	0.419	–1.986	0.140
Distance in Km=’30–44’	0.030	0.965	–0.392	0.739
Distance in Km=’45–59’	0.755	0.221	1.612	0.238
Number of Comorbidities	–0.029	0.806	0.032	0.553
Number of Admissions in the Last Year	0.073	**0.037**	0.105	**0.005**
Smoking Status=Smoker	0.948	0.157	0.609	0.574
Discharged Against Medical Advice=No	0.411	0.477	0.378	0.625
Compliant with Treatment=Not Compliant	–0.325	0.629	–0.734	0.387
Discharge Instructions= Incomplete	0.514	0.252	1.600	0.072
Follow-Up-Plan=Unclear	0.216	0.828	0.011	0.996
Discharge during Weekends or Weekdays=Weekends	–0.152	0.765	–0.449	0.658
Number Of Procedures	0.749	**0.000**	0.721	**0.000**
Delay (Time between Discharge and Readmission)	0.007	0.722	–0.006	0.853

The next step used t-tests to determine if there were differences as to: length of stay, readmission delay, age, number of co-morbidities, previous admissions, and the number of procedures with respect to gender, marital status, insurance status, whether patients were given clear follow-up plans and discharge instructions, if they were compliant with their treatment plans, and if they smoked. For all hospital readmissions (n=1157) the only gender difference was that females had a higher number of co-morbidities, and none of the factors had any impact on length of stay and readmission delay. Interestingly, coinsurance and smoking status showed statistically significant differences; age, number of previous admissions, and number of co-morbidities were significantly greater for smokers. As to insurance status, patients who paid less than 20% as a coinsurance had more previous admissions, while those whose co-pay was greater than 20% had a significantly greater number of medical procedures. Furthermore, patients who had more medical procedures were less compliant with medical treatment plans, and more likely to be discharged AMA (See Table 2). Clear discharge instructions did not vary between the groups, but those who had unclear follow-up plans had more previous admissions.

**Table 2:** All admission and readmission factors with significant t-test results.

**1- All Admissions N=3962**	T-Statistic Values (–/+) and Significance Level (*/**)					

*H*_0_ : *μ*_1_–*μ*_2_= 0 Regarding groups of	LOS	Age	Delay	Admissions-Year back	# Procedures	# Comorbidity
Gender (Male – Female)	–1.770	0.763	–0.997	–0.715	1.016	– 0.531
Smoking (Non – Yes)	1.300	–4.089**	0.034	–8.626**	0.307	–11.258**
Compliant with treatment (No – Yes)	1.331	0.871	–0.347	–1.393	1.760	0.066
Marital Status (Single – Married)	1.281	–43.564**	0.241	0.428	–4.247**	–18.468**
Discharge (Weekend – Weekday)	0.480	–2.529*	1.632	–0.465	– 0.040	– 0.930
Coinsurance (<20% – >20%)	2.449	–9.360**	–0.113	15.699**	–1.733	0.016
Discharge Decision (Not AMA – Against Medical)	–0.199	–0.842	0.315	–1.141	–1.821	–2.645
Follow-up plan (Unclear – Clear)	1.200	2.685	–0.993	1.063	1.407	3.176*
Discharge Instructions (Unclear – Clear)	0.600	0.664	–1.225	1.388	–0.138	0.625

**2- Readmissions N=1157**	T-Statistic Values (–/+) and Significance Level (*/**)					

*H*_0_ : *μ*_1_–*μ*_2_= 0 Regarding groups of	LOS	Age	Delay	Admissions-Year back	# Procedures	# Comorbidity
Gender (Male – Female)	–1.140	1.250	–0.997	–1.514	–0.023	–2.175*
Smoking (Non – Yes)	0.157	–3.798**	0.034	–4.394**	–0.045	–5.611**
Compliant with Treatment (No – Yes)	1.224	1.125	–0.866	–0.908	2.550*	–0.211
Marital Status (Single – Married)	1.921	–24.721**	0.241	0.034	–1.006	–11.279**
Discharge (Weekend – Weekday)	–0.216	–2.307	1.632	0.579	–0.381	0.779
Coinsurance (<20%–>20%)	1.264	–7.166**	–0.113	7.695**	–2.862**	–1.306
Discharge Decision (Not AMA – Against Medical)	–1.148	–1.251	0.315	1.064	–2.890*	–1.361
Follow-up plan (Clear –Unclear)	0.758	1.259	–0.993	–2.083*	0.912	0.589
Discharge Instructions (Clear – Unclear)	1.454	0.807	–1.225	–1.614	1.313	– 0.252

(*): Significant at 0.05, (**): Significant at 0.001.

For avoidable readmissions, patients who had unclear discharge instructions had more co-morbidities (See Table 3), and there were statistically significant differences in regards to marital status and insurance. Like with readmissions, those with a less than 20% of coinsurance were more likely to have more previous admissions. Furthermore, those that were married were older and had more co-morbidities. This was in contrast to singles who had a longer length of stay and more previous admissions. As to smoking, it was statistically significant in relation to higher number of co-morbidities.

**Table 3:** Avoidable readmission factors with significant t-test results.

Avoidable readmissions N=506	T-Statistic Values (–/+) and Significance Level (*/**)					

*H*_0_ : *μ*_1_–*μ*_2_= 0 Regarding groups of	LOS	Age	Delay	Admissions-Year back	# Procedures	# Comorbidity
Gender (Male – Female)	–2.420*	–0.466	–0.927	1.463	–1.094	–0.999
Smoking (Non – Yes)	0.113	–1.259	0.557	–1.845	0.603	–2.544*
Compliant with Treatment (No – Yes)	0.388	–0.358	–0.778	–0.139	1.339	–1.738
Marital Status (Single – Married)	2.461*	–17.935**	0.141	2.298 *	–0.705	–8.047**
Discharge (Weekend – Other)	–0.212	–2.779	1.446	0.606	–0.232	–0.585
Coinsurance (<20%–>20%)	1.256	–5.146**	–0.929	4.900**	–0.978	–0.640
Discharge Decision (Not AMA – Against Medical)	–0.781	0.286	0.183	–0.568	–1.899	– 0.228
Follow-up plan (Clear – Unclear)	–0.836	0.633	–0.238	–1.379	– 0.795	–1.125
Discharge Instructions (Clear – Unclear)	0.525	–1.247	–0.377	–1.383	–0.972	–3.640**

(*): Significant at 0.05, (**): Significant at 0.001.

Although the t-tests did not show a clear difference in relation to discharge instructions and follow-up plans, the use of data mining clearly established their importance (see Table 4). Using the JRip rule-based classifier model (with a ROC area = 0.71 and accuracy rate ≥ 75%), gave us a clearer picture of early hospital readmission. This model performed as well or better than most readmission prediction models proposed previously [14,17,83], and similar or better than other internal medicine studies [9,15,16,39]. Additionally, very few prediction models have been developed to examine potentially avoidable readmissions as an outcome [23,84,85].

**Table 4:** JRip data mining rules for avoidable readmissions.

**Rules for all readmission (readmissions vs. no readmissions)** ROC area= 0.71 and Accuracy > 75%	**Predicted class**	**Accuracy**

**Rule 1**: (MAIN_DISEASE_CAT = ICD-10 CAT 2) and (ADMISIONS_AYEAR_BACK >= 5)	Readmission	74%
**Rule 2:** (MAIN_DISEASE_CAT = ICD-10 CAT 2)	Readmission	69%
**Rule 3:** (ADMISIONS_AYEAR_BACK >= 2, (DISCHARGE_INSTRUCTIONS = no evidence of clear), and (AGE <= 65)	Readmission	73%
**Rule 4:** (ADMISIONS_AYEAR_BACK >= 3) and (DISCHARGE_DECISION = against medical advice)	Readmission	77%
**Rule 5:** (COINSURANCE = <20), (FOLLOW_UP_PLAN = no evidence of clear), (MED_COMPLIANT = not compliant with treatment), and (ADMISIONS_AYEAR_BACK >= 1)	Readmission	94%
**Rule 6:** (ADMISIONS_AYEAR_BACK >= 2), (DISCHARGE_INSTRUCTIONS = no evidence of clear), (NUMBER_OF_PROCEDURES <= 1), (NUM_COMORBIDITY <= 3), and (AGE >= 33)	Readmission	82%

**Rules of potentially avoidable readmissions (avoidable vs. unavoidable)**	**Predicted class**	**Accuracy**

**Rule 1:** (AGE >= 61), (FOLLOW_UP_PLAN = no evidence of clear), and (MAIN_DISEASE_CAT = ICD-10 CAT 10)	Avoidable	83%
**Rule 2:** (AGE >= 61) and (DISCHARGE_DECISION = against medical advice)	Avoidable	89%
**Rule 3:** (MED_COMPLIANT = not compliant)	Avoidable	100%
**Rule 4:** (FOLLOW_UP_PLAN = no evidence of clear), (AGE >= 56), (NUMBER_OF_PROCEDURES >= 9) and (NUMBER_OF_PROCEDURES <= 14)	Avoidable	84%
**Rule 5:** (FOLLOW_UP_PLAN = no evidence of clear), (AGE >= 61), (NUMBER_OF_PROCEDURES <= 4), (DELAY <= 10), and (LOS >= 3)	Avoidable	84%
**Rule 6:** (DISCHARGE_INSTRUCTIONS = no evidence of clear), (DELAY > 12), (DELAY < 16), and (NUM_COMORBIDITY >= 3)	Avoidable	75%
**Rule 7:** (FOLLOW_UP_PLAN = no evidence of clear), (SMOKING STATUS = smoker), and (ADMISIONS_AYEAR_BACK <= 3)	Avoidable	79%
**Rule 8:** (DISCHARGE_DECISION = against medical advice)	Avoidable	86%
**Rule 9:** (FOLLOW_UP_PLAN = no evidence of clear), (MAIN_DISEASE_CAT = ICD-10 CAT 5), and (AGE <= 57)	Avoidable	79%

**Abbreviations:** ICD-10 CAT 2: Malignant Neoplasms. ICD-10 CAT 5: Endocrine, nutritional and metabolic diseases. ICD-10 CAT 10: Diseases of the circulatory system.

For each analysis all readmitted patients were compared with those not readmitted. Then for all readmissions, unavoidable readmissions were compared with those identified as avoidable readmissions Table 4 shows that patients who were having < 20% coinsurance, had no clear follow-up plan, were non-compliant with their prescribed regimen, and who had one or more admissions in the last year were at an extremely high risk of readmission, 94%. While adults (33 year or older) who had been readmitted two or more times in the past year, with three or fewer co-morbidities, only having had one procedure at their initial admission but had not received a clear discharge care plan, had an 82% chance of being readmitted. Thus, the data mining clearly linked various risk factors together resulting in high risk probabilities of readmission.

For avoidable readmissions, the model showed that all patients (100%) who did not adhere with their medical treatment regimen had a preventable readmission. Additionally, patients who left AMA had an 86% chance of readmission, and this increased to 89% for those over 61. Moreover, patients aged 61+ with circulatory system related diseases and no clear follow-up plan had an 83% chance of being avoidably readmitted. This risk was reduced slightly for patients with endocrine, nutritional, and metabolic diseases that were over age 56. Patients also had a 79% chance of being readmitted when they did not have a clear discharge plan, as did smokers with fewer than three admissions and no clear follow-up plan. Finally, the model showed that patients with three or more co-morbidities, no clear discharge instructions, and a readmission delay period between 12 and 16 days had a 75% risk of avoidable readmission (See Table 4).

Data mining tools provided a set of clear rules that could be used to determine probability of avoidable readmission, but it did not isolate the impact of individual factors independently. Therefore, in the last stage of analysis a logistic regression with all the identified factors was completed (See Table 5). By determining the odds ratio, the actual impact of each risk was determined using all hospital readmissions and then the subset of avoidable hospital readmissions.

**Table 5:** Logistic regression model of factors influencing all readmissions & avoidable readmissions.

		All Readmissions		Avoidable Readmission	

Factor	Levels	Adjusted Odd’s Ratio	95% Confidence Interval	Adjusted Odd’s Ratio	95% Confidence Interval
Gender	Female Male	0.971	0.847–1.114	0.879	0.729–1.061
Smoking	Yes No	2.318	1.953–.751*	1.816	1.393–2.367*
Compliant	Yes No	0.054	0.0270.109*	0.760	0.470–1.230
Marital status	Married Single	0.981	0.815–1.181	0.963	0.749–1.238
Discharge	Weekday Weekend	1.037	0.827–1.300	1.317	0.943–1.838
Coinsurance	≥20% <20%	0.393	0.032–.047*	1.617	1.145–2.282*
Discharge Decision	Not AMA AMA	0.247	0.1750.349*	0.106	0.057–0.197*
Follow-up plan	Clear Unclear	0.662	0.565–0.776*	0.338	0.249–0.460*

Discharge Instruction	Clear Unclear	0.633	0.565–0.779*	0.337	0.277–0.512*
Age		1.096	0.994–1.111*	1.019	1.012–1.025*
LOS		1.166	1.143–1.189*	1.027	1.008–1.046*
Delay		NA	–	0.998	0.982–1.013
Adm. Year Back		1.022	1.010–1.034*	0.963	0.939–0.989*
Number of procedures		0.992	0.981–1.003	1.043	1.021–1.065*
Number of co morbidity		1.109	1.069–1.150*	1.196	1.124–1.272*

(*): Significant at 0.05.

For all readmissions the one dichotomous risk factor that increased one’s odds of being readmitted was smoking (OR= 2.318, 95% CI 1.953–2.751), which should have been anticipated, due to established health risks as well as the linkage to various co-morbidities. The probability of readmission was increased by 131.8% for smokers. Interestingly enough, five of the factors decreased one’s risk of readmission: patient compliance with medical treatment (OR= 0.054, 95% CI 0.027–0.109), patients receiving a clear follow-up plan and clear discharge instruction (OR= 0.662, 95% CI 0.565– 0.776; OR= 0.633, 95% CI 0.565–0.779, respectively), patients being properly discharged by the physician (OR= 0.247, 95% CI 0.175–0.349), and having a 20% or more as a coinsurance (OR= 0.393, 95% CI 0.032–0.047). Therefore, those who were compliant with their medical treatment would have readmission with lower probability (0.054%) comparing with those who were not compliant with their treatment plans. Similarly, the probability of readmission decreased by 75% for those who followed their physicians’ advice and discharge date. Having a clear medical follow-up plan could reduce readmission by 34%, and having clear discharge instructions would reduce the likelihood of readmission by 37%. Finally, if the patient was responsible for a 20% of coinsurance, the readmission rate would be decreased by 61% (See Table 5).

Many of the factors had similar results for avoidable admissions as well. Smoking increased one’s risk of readmission by 1.816 times (OR 1.816, 95% CI 1.393–2.367), while having a clear follow-up plan and clear discharge instructions greatly reduced the likelihood of avoidable readmission (OR 0.338, 95%; CI 0.249–0.446; OR 0.337, 95% CI 0.227–0.512, respectively), as did being discharged following physician’s guidance, OR 0.106, 95% CI 0.057–0.197. Therefore, having effective discharge instructions could reduce the risk of avoidable readmissions by 64%. Similarly, those who had clear follow-up plans had a lower chance of avoidable readmission (34%) comparing with those who had unclear follow-up plans. However, if patients had a coinsurance of 20% or more they were more likely to have an avoidable readmission (OR 1.617, 95% CI 1.145–2.282).

The logistic regression supported the previous findings from the multiple regression model in regards to: length of stay, number of previous admissions, and age (OR = 1.166, 1.022, and 1.069, respectively, p <0.01) for all readmissions, with similar findings for avoidable readmissions (length of stay OR =1.027; previous admissions OR 0.963; and age OR = 1.019, p<0.01). In addition, the number of co-morbidities (OR = 1.196) and previous number of procedures (OR =1.043) were statistically significant, p<0.01, for avoidable readmissions.

Thus the significant factors from the multiple linear regression, the t-tests and the data mining all were identified as independently significant risk predictors of hospital readmission with the logistic regression. Moreover, many of these were modifiable behavioural factors that can be addressed through patient education and behaviour modification, changes in physician knowledge integrating EBM, or through hospital policy.

## Discussion/Implications

Hospital readmissions can be reduced by identifying the attributes of patients at higher risk of readmission. Unlike previous studies that tried to identify risk factors associated with readmission [9,14,15,17,42], the current study used several different methods of analysis and adopted an evidence based approach to better gain insight into which factors could be consistently identified as modifiable and that could be easily interpreted by clinicians to help them to take proper action, resulting in better patient outcomes and a reduction in avoidable hospital readmissions.

**Figure 2:**
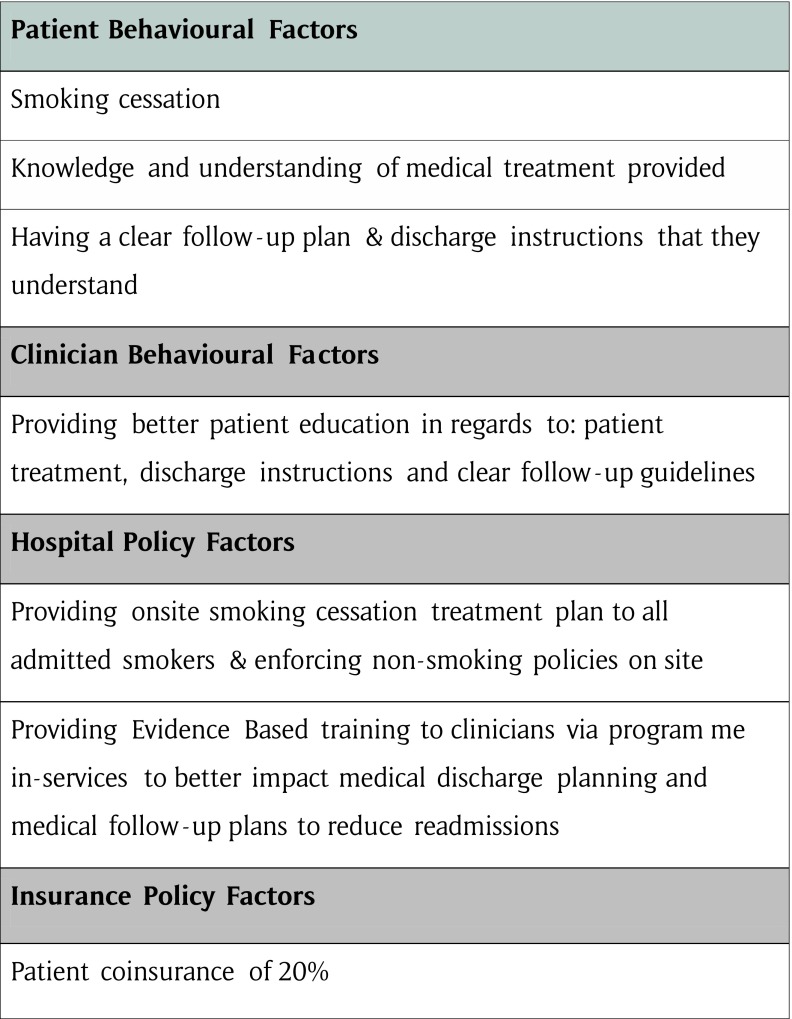
Heuristic guide impacting hospital readmissions.

From our findings prior hospital admissions, discharge against medical advice, having health insurance coverage with a coinsurance less than 20%, receiving no clear follow-up plan or discharge instructions, being non-compliance with their treatment, the number of procedures in the index admission, smoking, the number of co-morbidities, and patient age all had an impact on 30-day hospital readmissions. It is obvious that the risk factors of all readmissions, except insurance status, were predictors of potentially avoidable readmissions, as well. Therefore, efforts to reduce early readmissions should be effectively directed towards avoidable cases. Given the impact of avoidable readmission rates on hospitals [23,27], it is important for hospitals to differentiate between modifiable and non-modifiable risk factors [20]. Modifiable risk factors would yield a higher response rate (See Figure 2). Although age is not modifiable, many other risk factors are clearly modifiable with minimal costs, and implementing evidence based health programmes that modify patient and clinical provider behaviours leading to a 30% reduction in readmissions could result in hundreds of thousands in saving for countries where the government is paying the majority of the costs.

Smoking is clearly a modifiable risk factor, but while most hospitals in Jordan have a non-smoking policy, they are not actively implemented, therefore hospital policies should be targeted with evidence based education programmes to staff and administration on the benefits of a clean air policy. A supportive smoking cessation programme should be implemented when a smoker is first admitted, with continued programme support after discharge, as part of the care continuum. This is important in developing countries especially where smoking is still an accepted behaviour, among men. As the WHO data (2015) indicates, almost half of all men (47%) older than 15 are smokers in Jordan [73].

It is also clear that non-compliance with one’s treatment regimen and leaving AMA are the strong predictors of avoidable readmissions. Development of and implementation of programmes based on Prochaska’s stages of change [66] could be focused on pre-contemplation and contemplation by increasing patient awareness and enhancing communication with patients and their families—targeting at risk patients —as they are unaware of the implications these choices have on their health (See Figure 2).

Data mining tools such as JRip rules can be used to identify behavioural risk factors and to track those patients who are at higher risk of readmission. For example, patients who were discharged against medical advice need to be warned out that they are at higher risk of being avoidably readmitted to the hospital. Also, patient medical compliance history could be obtained from historical data (if available) of previous admissions. This urges the need for having a national healthcare database were healthcare organisations report patient behaviour characteristics to benefit predicting future hospital admissions.

From the clinician’s side, much can be done, for 91% of physicians surveyed in a Jordan study believed that adopting EBM practices would be beneficial, however the majority did not feel comfortable with adopting or using EBM theory in practice [86]. Therefore first and foremost programmes should be conducted to help physicians understand the benefits of using EBM, terms and epidemiological measures often used (e.g. PICO, relative risk and population risk reduction, attributable risk, etc.) and how to integrate these findings into their practices, in order to reduce hospital readmissions. In this regard, obtained JRip rules can be implemented as a DSS in hospitals to predict mode of readmission. These rules can be embedded in the system to integrate factors related to EBM and evidence based public health to better predict avoidable readmissions. For instance, in order to take advantage of the JRip rules to reduce avoidable readmissions, hospital policies regarding EBM must be revised to force physicians to provide clear discharge instructions and follow-up plan.

Better training should also be provided to help them understand the positive impact of clear follow-up instructions and clearly understood discharge treatment plan in patient outcomes and reducing readmissions [50,51,52,87]. Auerbach and colleagues [84] found that among the most common predictors associated with avoidable readmissions were follow-up appointment not scheduled clearly post-discharge, and patients’ lack of knowledge of whom to contact and where to go after discharge. Clear discharge plans, such as providing patients with education and instructions including: safe and effective use of all medications and medical technology, diet and nutrition, pain management, and rehabilitation techniques. Whereas, follow-up instructions should reflect cooperation within the hospital units and between healthcare practitioners, and outside providers to ensure timely care and integrated services. Hospital guidelines for discharge planning and follow-ups should then be monitored using tools such as DSS and data mining techniques to determine the impact of these protocols on avoidable readmissions. Early follow-up and continuity of healthcare are effective interventions for preventing readmission [50,88]. Finally, insurance structures need to be considered, even though it may be seen as a moral hazard, it is clearly evident that avoidable hospital readmissions predominately occur when the patient has more coinsurance, or financial responsibility for their health. Thus, hospitals should develop policies and programmes that can address these risks, from both the patient and provider perspective.

Even though this study employed several techniques to validate the risk factors associated with hospital readmissions, the data was limited to one large public teaching hospital, in a developing country, so the generalizability may be limited in scope. Furthermore, we did not capture those patients who were readmitted to another hospital and those who received their treatment and follow-up from a primary healthcare centre or other healthcare facility; it was unclear what happened to them once they were discharged from the hospital. Despite these limitations, the study provides evidence based recommendations that are easy to understand and can be practically interpreted by caregivers and other stakeholders, including patients and administrative staff.

## Conclusion

This study addresses several gaps of previous research and adds to a growing understanding of the interrelated risk factors associated with deemed avoidable hospital readmission. Hospitals could integrate EBM with behavioural health programmes in their policies for patients and clinicians, as a potential way of reducing hospital readmissions, where a significant portion of these readmissions could be prevented.

However, further research is still needed and more focus should be directed toward developing effective evidence based programmes to control readmission rates.

## Supplementary Files

The supplementary files for this article can be found as follows:

- **Supplementary File 1:** Tables. ijic-16-3-2436-s1.pdf
