# Supplementary material for: Integrating the Principles of Evidence Based Medicine and Evidence Based Public Health: Impact on the Quality of Patient Care and Hospital Readmission Rates in Jordan
Source: Int J Integr Care. 2016 Aug 31;16(3):12. doi: 10.5334/ijic.2436 (PMC5388041; doi:10.5334/ijic.2436)
Supplement: Supplementary file 1 [file ijic-16-3-2436-s1.pdf]

Appendix I. The most frequent procedures among index admissions for cases that were readmitted to the hospital.

	All Readmissions			Avoidable Readmissions		
**Disease**	Procedure	Frequ-ency	Valid Percent	Procedure	Frequ-ency	Valid Percent
**Cerebrovascular Diseases**	X-ray chest one view	10	13.33	Left heart cardiac catheterization	5	10.20
	Diagnostic ultrasound of heart	8	10.67	X-ray chest one view	5	10.20
	Tomography of head	6	8.00	Diagnostic ultrasound of heart	5	10.20
	Left heart cardiac catheterization	5	6.67	Tomography of head	4	8.16
	Combined physical therapy without mention of the components	5	6.67	Other transfusion of whole blood	4	8.16
	Magnetic resonance imaging of brain and brain stem	4	5.33	Diagnostic ultrasound of peripheral vascular system	3	6.12
	Electrocardiogram	4	5.33	Magnetic resonance imaging of brain and brain stem	3	6.12
	Other transfusion of whole blood	4	5.33	Arteriography of renal arteries	2	4.08
	Diagnostic ultrasound of abdomen and retroperitoneum	3	4.00	Diagnostic ultrasound of abdomen and retroperitoneum	2	4.08
	Diagnostic ultrasound of peripheral vascular system	3	4.00	Initial insertion of single-chamber device	1	2.04

	All Readmissions			Avoidable Readmissions		
**Disease**	Procedure	Frequ-ency	Valid Percent	Procedure	Frequ-ency	Valid Percent
**Chronic Lower Respiratory Diseases**	X-ray chest one view	39	28.06	X-ray chest one view	31	36.47
	Combined physical therapy without mention of the components	12	8.63	Respiratory medication administered by nebulizer	6	7.06
	Respiratory medication administered by nebulizer	10	7.19	Hemodialysis	4	4.71
	Diagnostic ultrasound of heart	8	5.76	Diagnostic ultrasound of heart	4	4.71
	Electrocardiogram	7	5.04	Diagnostic ultrasound of urinary system	3	3.53
	Cardiac tomogram	5	3.60	Electrocardiogram	3	3.53
	Hemodialysis	4	2.88	Cardiac tomogram	2	2.35
	tomography of head	4	2.88	Diagnostic ultrasound of abdomen and retroperitoneum	2	2.35
	Diagnostic ultrasound of urinary system	3	2.16	Electroencephalogr am	2	2.35
	Diagnostic ultrasound of peripheral vascular system	3	2.16	Infusion of immunosuppressiv e antibody therapy	1	1.18

Disease	All Readmissions			Avoidable Readmissions		
	Procedure	Frequ-ency	Valid Percent	Procedure	Frequ-ency	Valid Percent
Diabetes Mellitus	X-ray chest one view	32	18.29	X-ray chest one view	14	12.28
	Respiratory medication administered by nebulizer	15	8.57	Diagnostic ultrasound of heart	9	7.89
	Diagnostic ultrasound of heart	12	6.86	Porcine wound dressing	8	7.02
	Electrocardiogram	12	6.86	Excisional debridement of wound, infection, or burn	7	6.14
	Porcine wound dressing	8	4.57	Electrocardiogram	7	6.14
	Excisional debridement of wound, infection, or burn	7	4.00	Tomography of head	6	5.26
	Tomography of head	7	4.00	Other chest x-ray	4	3.51
	Diagnostic ultrasound of abdomen and retroperitoneum	5	2.86	Diagnostic ultrasound of abdomen and retroperitoneum	4	3.51
	Other chest x-ray	4	2.29	Magnetic resonance imaging of spinal canal	4	3.51
	Magnetic resonance imaging of spinal canal	4	2.29	Hemodialysis	3	2.63

	All Readmissions			Avoidable Readmissions		
**Disease**	Procedure	Frequ-ency	Valid Percent	Procedure	Frequ-ency	Valid Percent
**Ischaemic Heart Diseases**	X-ray chest one view	72	22.78	X-ray chest one view	52	24.30
	Diagnostic ultrasound of heart	27	8.54	Diagnostic ultrasound of heart	17	7.94
	Respiratory medication administered by nebulizer	26	8.23	Respiratory medication administered by nebulizer	17	7.94
	Electrocardiogram	19	6.01	Electrocardiogram	10	4.67
	Tomography of head	13	4.11	Hemodialysis	9	4.21
	Hemodialysis	10	3.16	Tomography of head	9	4.21
	Combined physical therapy without mention of the components	10	3.16	Daily living activities therapy	6	2.80
	Cardiac tomogram	8	2.53	Insertion of endotracheal tube	6	2.80
	Insertion of endotracheal tube	8	2.53	Left heart cardiac catheterization	5	2.34
	Left heart cardiac catheterization	7	2.22	Cardiac tomogram	5	2.34

	All Readmissions			Avoidable Readmission		
**Disease**	Procedure	Frequ-ency	Valid Percent	Procedure	Frequ-ency	Valid Percent
**Malignant Neoplasms**	X-ray chest one view	413	21.11	X-ray chest one view	90	21.18
	Electrocardiogram	160	8.18	Electrocardiogram	31	7.29

	Diagnostic ultrasound of heart	141	7.21	Diagnostic ultrasound of heart	30	7.06
	Combined physical therapy without mention of the components	82	4.19	Combined physical therapy without mention of the components	29	6.82
	Respiratory medication administered by nebulizer	75	3.83	Other transfusion of whole blood	27	6.35
	Other transfusion of whole blood	63	3.22	Magnetic resonance imaging of brain and brain stem	14	3.29
	Tomography of head	62	3.17	Tomography of head	11	2.59
	Injection or infusion of cancer chemotherapeutic substance	59	3.02	Injection or infusion of cancer chemotherapeutic substance	10	2.35
	Magnetic resonance imaging of brain and brain stem	39	1.99	Diagnostic ultrasound of urinary system	9	2.12
	Diagnostic ultrasound of abdomen and retroperitoneum	36	1.84	Magnetic resonance imaging of spinal canal	8	1.88

	**All Readmissions**			**Avoidable Readmissions**		
**Disease**	Procedure	Frequ-ency	Valid Percent	Procedure	Frequ-ency	Valid Percent
**Other Forms Of Heart Disease**	X-ray chest one view	20	21.51	X-ray chest one view	9	21.43
	Diagnostic ultrasound of heart	7	7.53	Magnetic resonance imaging of spinal canal	6	14.29

	Hemodialysis	6	6.45	Diagnostic ultrasound of heart	4	9.52
	Diagnostic ultrasound of abdomen and retroperitoneum	6	6.45	Infusion of immunosuppressive antibody therapy	3	7.14
	Magnetic resonance imaging of spinal canal	6	6.45	Hemodialysis	2	4.76
	Infusion of immunosuppressive antibody therapy	4	4.30	Transabdominal gastroscopy	2	4.76
	Transabdominal gastroscopy	4	4.30	Tomography of head	2	4.76
	Endovascular repair or occlusion of head and neck vessels	3	3.23	Diagnostic ultrasound of urinary system	2	4.76
	Electrocardiogram	3	3.23	Electrocardiogram	2	4.76
	Percutaneous angioplasty or atherectomy of precerebral (extracranial) vessel(s)	2	2.15	Endoscopy without biopsy	1	2.38

	**All Readmissions**			**Avoidable Readmissions**		
**Disease**	Procedure	Frequ-ency	Valid Percent	Procedure	Frequ-ency	Valid Percent
**Renal Failure**	X-ray chest one view	29	21.48	X-ray chest one view	10	18.52
	Combined physical therapy without mention of the components	15	11.11	Diagnostic ultrasound of heart	5	9.26
	tomography of head	11	8.15	Magnetic resonance imaging of brain and brain stem	4	7.41

	Diagnostic ultrasound of heart	9	6.67	Injection or infusion of other therapeutic or prophylactic substance	4	7.41
	Magnetic resonance imaging of brain and brain stem	7	5.19	Intravascular imaging of extracranial cerebral vessels	2	3.70
	Hemodialysis	5	3.70	Hemodialysis	2	3.70
	Computerized axial tomography of abdomen	5	3.70	Tomography of head	2	3.70
	Electrocardiogram	4	2.96	Computerized axial tomography of abdomen	2	3.70
	Injection or infusion of other therapeutic or prophylactic substance	4	2.96	Diagnostic ultrasound of urinary system	2	3.70
	Computerized axial tomography of thorax	3	2.22	Respiratory medication administered by nebulizer	2	3.70
